# Draft macronuclear genome sequence of the marine ciliate *Miamiensis avidus* causing flatfish scuticociliatosis

**DOI:** 10.1128/mra.00412-24

**Published:** 2025-03-28

**Authors:** Na Young Kim, Sang Jung Ahn, Eun Ji Jeon, Moo-Sang Kim, Mi Young Cho

**Affiliations:** 1 Pathology Research Division, National Institute of Fisheries Science, Busan, South Korea; 2 Certification & Evaluation Team, Korea Institute of Marine Science & Technology Promotion, Seoul, South Korea; 3 Department of Bioinformatics, thMOAGEN, Daejeon, South Korea; University of California Riverside, Riverside, California, USA

**Keywords:** scuticociliatosis, *Miamiensis avidus*, genome sequence, flatfish

## Abstract

Scuticociliatosis is a severe and challenging protozoal disease affecting farmed flatfish and crustaceans. This draft genome assembly of the macronuclear (MAC) genome of *Miamiensis avidus*, a scuticociliate parasite, will be valuable for understanding the somatic functions, physiology, and pathology of scuticociliates in aquaculture worldwide.

## ANNOUNCEMENT

*Miamiensis avidus* is a free-living marine protozoan belonging to the order Philasterida, subclass Scuticociliatida, class Oligohymenophorea, and phylum Ciliophora. It has caused severe disease outbreaks and mass mortalities in cultured marine fish such as turbot (1), sea bass (2), and olive flounder (*Paralichthys olivaceus*) (3), but an effective chemical treatment for this disease has not yet been discovered. Therefore, we sequenced the macronuclear (MAC) genome of a survey for pandemic serotypes from *M. avidus* to help us understand its genomic and biological features and the development of an effective vaccine against parasitic disease.

*M. avidus* strain MagSerotype2-33 (*M. avidus* Serotype 2, SCU-MA2) was isolated from olive flounder *P. olivaceus* on a local farm in Pohang, Korea, in 2008. To obtain macronuclear DNA, *M. avidus* strain SCU-MA2 was cultured in the CHSE-214 cell line (ATCC CRL 1691) at 20°C in Eagle’s MEM with 10% heat-inactivated fetal bovine serum (FBS). Macronuclei were isolated from the cell lysate using a Whatman 25 mm Nuclepore Polycarbonate filter membrane (pore size: 5 µm), as previously reported (4). *M. avidus* MAC DNA was extracted using a HiGene Genomic DNA Prep Kit (Biofact, Korea). The sample was used to prepare the SMRTbell library with an SMRTbell Template Prep Kit. Sequencing was performed on the PacBio sequencing platform (Pacific Biosciences, USA). Additionally, MiSeq sequencing data previously reported and used for the assembly of the complete mitochondrial genome of this species were also used to polish the *M. avidus* genome sequence (5).

The sequencing of the PacBio SMRT cell produced 22.3 Gbp of raw reads at 293.6-fold coverage of the genome. The reads were assembled using CANU v. 1.6 with default parameters (6). To enhance the accuracy of the initial contig assembly, clean Illumina short reads were aligned using BWA-MEM (7), followed by polishing with Pilon version 1.23 (8), both executed with default parameters. The resulting assembly was searched for putative telomeric repeats (5′-CCCCAA)n using BioEdit and identified 119 contigs with telomeric repeat sequences on both ends and 49 with only a single telomere found. The final draft *M. avidus* MAC genome was assembled into 213 contigs with a total sequence length of 75,782,695 bp and a contig N50 of 754,125 bp (L50: 30, 23.52% GC content). The annotation information derived from the draft genome contig sequences, along with the corresponding transcript and protein sequences, has been deposited in “https://doi.org/10.6084/m9.figshare.26866936.v2.” By combining the information and comparisons between the *M. avidus* MAC genome and those of six other ciliate species, *M. avidus* was found to be closely related to *Pseudocohnilembus persalinus* as a member of the Order Philasterida (Table 1). The use of the draft MAC genome could have widespread implications for gaining a better understanding of the pathology of scuticociliatosis, as well as for developing effective treatment strategies and fish vaccines.

**TABLE 1:** Comparison of different ciliate macronuclear genomes with *M. avidus* MAC genome

Parameters	*Miamiensis avidus*	*Pseudocohnilembus persalinus*	*Paramecium bursaria*	*Paramecium caudatum*	*Paramecium tetraurelia*	*Tetrahymena thermophila*	*Oxytricha trifallax*
**Genome size (Mb**)	75.8	55.5	26.8	30.5	72.07	103.01	55.4
**Sequencing platform**	PacBio/Illumina	Illumina	PacBio/Illumina	Illumina	Sanger/Illumina	Sanger/Illumina	PacBio/Illumina
**No. of contigs**	213	288	515	1,202	697	1,158	19,152
**Average GC content (%)**	23.5	19	28.8	28.2	28.0	22.0	31.0
**N50 (Kb)**	754	368	100	312.9	413	520	3.5
**Gene number**	10,723	13,186	15,101	18,673	40,460	26,996	18,400

## Data Availability

The PacBio raw data used to complete the draft MAC genome of *M*. *avidus* strain MagSerotype2-33 (*Miamiensis avidus* Serotype 2, SCU-MA2) have been uploaded to the NCBI Sequence Read Archive (SRA) database under accession number SRR30663476. Additionally, the assembly of the draft MAC genome sequences of this *M. avidus* has been registered in GenBank under accession number GCA_037039885.1, and the scaffold contig sequences have been registered in the NCBI GenBank under JAWVGU010000000.
